# Measuring disability-adjusted life years (DALYs) due to low back pain in Malta

**DOI:** 10.1186/s13690-020-00451-w

**Published:** 2020-07-23

**Authors:** Sarah Cuschieri, Grant M. A. Wyper, Neville Calleja, Vanessa Gorasso, Brecht Devleesschauwer

**Affiliations:** 1 Department of Anatomy, Faculty of Medicine and Surgery, University of Malta, Msida, Malta; 2 Public Health Scotland, Meridian Court, 5 Cadogan Street, Glasgow, G2 6QE Scotland; 3 Department of Public Health, Faculty of Medicine and Surgery, University of Malta, Msida, Malta; 4 Director of Directorate of Health Information and Research, Ministry of Health, Gwardamangia, Malta; 5 Department of Epidemiology and Public Health, Sciensano, Brussels, Belgium; 6 Department of Public Health and Primary Care, Ghent University, Ghent, Belgium; 7 Department of Veterinary Public Health and Food Safety, Ghent University, Merelbeke, Belgium

**Keywords:** Low back pain, Epidemiology, Burden, Outcome research, Malta, Burden of disease, YLL, YLD, DALYs, GBD, European burden of disease network

## Abstract

**Background:**

Low back pain (LBP) is a public health concern and a leading cause of ill health. A high prevalence of musculoskeletal complaints has been reported for Malta, a small European state. The aim was to estimate for the first time the burden of LBP at population level in Malta in terms of disability-adjusted life years (DALYs) and compare to estimates obtained by the Global Burden of Disease (GBD) study.

**Method:**

The Maltese European Health Interview Survey dataset for 2015 provided the LBP prevalence data through representative self-reported history of chronic LBP within the past 12 months in combination with limitations to daily activities. Proportions of LBP severity (with and without leg pain – mild, moderate, severe and most severe) and their corresponding disability weights followed values reported in the GBD study. Years lived with disability (YLD) for LBP were estimated for the whole population by age and sex. Since LBP does not carry any mortality, YLD reflected DALYs. The estimated local DALYs per 100,000 were compared to the GBD 2017 study results for Malta for the same year.

**Results:**

LBP with activity limitation gave a point prevalence of 6.4% (95% Uncertainty Interval [UI] 5.7–7.2%) (5.6% males [95% UI 4.6–6.6%]; 7.3% females [95% UI 6.2–8.4%]), contributing to a total of 23,649 (95% UI 20,974–26,463) Maltese suffering from LBP. The LBP DALYs were of 716 (95% UI 558–896) per 100,000. Females experienced higher LBP burden (739 [95% UI 575–927] DALYs per 100,000) than males (693 [95% UI 541–867] DALYs per 100,000). Our DALY estimates were lower than those reported by the GBD 2017 study (i.e., 1829 [95% UI 1300–2466] per 100,000).

**Conclusions:**

LBP imposes a substantial burden on the Maltese population. Differences observed between national estimates and those of the GBD study suggest the integration of updated locally sourced data into the model and encouraging local contributors in order to improve the DALY estimates of each country.

## Background

Low back pain (LBP) is a common health problem that causes limitations to daily activities as well as absence from work [1]. Furthermore, it creates a substantial burden on the economy at an individual, community, health care and population level [2, 3]. In fact, LBP ranks as the leading cause of years lived with disability (YLD) in 65% of the countries worldwide, and is the leading cause of YLD in all high income, and European, countries [4]. The situation is not different for the European country of Malta, a small state situated in the middle of the Mediterranean Sea with a residential population of approximately half a million. It has been reported that the commonest complaints encountered in primary health care services are due to musculoskeletal problems, with a predominance for LBP [5]. A similar situation is present for the orthopaedic outpatient cases that visit Mater Dei Hospital, which is the only state general hospital in Malta [6]. However, the national burden of LBP in terms of Disability-Adjusted Life Years (DALYs) has never been estimated at a population level in Malta. DALYs are a standard metric used to quantify burden in terms of morbidity and mortality [7]. The primary aim of the study was to estimate the national burden of disease at a population level. These estimates were then compared to those reported by the Global Burden of Disease (GBD) study for Malta for benchmarking purposes.

## Methods

### Data sources

The Maltese European Health Interview Survey (EHIS 2014–2015) dataset was used to establish the prevalence of LBP in Malta. The sample population was composed of a random single staged stratified population by age (15 years and above), sex and geographical districts. The prevalence of LBP was assumed to be 0% below the age of 15 years. The study population was representative of the Maltese population, with a survey response rate of 52% [8]. A weighting factor was applied to each individual in order to create representative population level estimates from the survey sample. The total adjusted population under study was of 4085 individuals (*n* = 2032 males) which represented 1% of the total Maltese population [8]. The Maltese population demographic data were obtained from the National Statistic annual report [9]. This was used to measure the prevalence of LBP at a population level by age-group and sex. The data source (Malta - EHIS) that was used to obtain the LBP prevalence by age-group and sex, combined the population aged 75 years and above as one entity. Hence, it was assumed that the prevalence of the study population making up the 75+ age-group was the same for the succeeding 5-year age-groups up till 90 years. The percentage was multiplied by the total Maltese population in each 5-year age-group (beyond 75 years) according to the National Statistics annual report. This gave an estimate of the Maltese adults suffering from LBP by 5-years age-group from 75 years to 90 years.

The LBP burden of disease model used in this study followed the same model that was used by the GBD 2017 study [4]. The data was analysed using IBM SPSS® version 21 for Mac and Microsoft® Excel version 16 for Mac and R version 3.6.1 [10].

### Definitions

The prevalence of LBP was defined as those individuals that reported suffering from back pain in the last 12 months who also indicated a limitation in their daily activities in the Global Activity Limitation Indicator (GALI) instrument [11]. The pool of prevalent cases was then stratified by 5-year age-group and sex. These cases were furthermore sub-divided based on the GBD 2017 study health state model [4]. This involved using proportional allocation to assign the prevalent cases into different health states which reflected categorical groups of severity of LBP defined as mild, moderate, severe and most severe. These proportions and their corresponding disability weights followed those reported by the GBD 2017 study as seen in Tables 1 and 2 [4, 12]. According to the GBD 2017 study, LBP was defined as LBP with or without pain referred into one or both legs that lasts for at least 1 day. Following the GBD 2017 study protocol, this also involved a stratified split of health states between those that suffered LBP with leg pain, and those that suffered LBP without leg pain. In total the estimated number of prevalent cases of LBP was assigned to four different health states stratified by two sequelae, resulting in cases being distributed to eight different categories per age-group [4]. This stratification was applied to the Malta specific EHIS data source to establish the prevalent cases of LBP by each of the different health states and age-groups.

**Table 1:** Summary of the health status definitions, proportions and the associated disability weights (with corresponding 95% confidence intervals] CI]) used for this study as reported by the GBD2017 study

Health State	Proportion	Disability weight (CI)
Overall low back pain	100%	N/A
Low back pain with leg pain	(Table 2)	
Low back pain without leg pain	(Table 2)	
Low back pain with leg pain, mild	27%	0.02 (0.01–0.04)
Low back pain with leg pain, moderate	36%	0.05 (0.04–0.08)
Low back pain with leg pain, severe	14%	0.33 (0.22–0.45)
Low back pain with leg pain, most severe	23%	0.38 (0.26–0.52)
Low back pain without leg pain, mild	41%	0.02 (0.01–0.04)
Low back pain without leg pain, moderate	35%	0.05 (0.04–0.08)
Low back pain without leg pain, severe	10%	0.27 (0.18–0.37)
Low back pain without leg pain, most severe	14%	0.37 (0.25–0.51)

**Table 2:** Proportions of individuals with low back pain and leg pain according to age groups as reported by the GBD2017 study

Age Group	Proportion (%) without leg pain	Proportion (%) with leg pain
15–19	84%	16%
20–24	77%	23%
25–29	71%	29%
30–34	69%	31%
35–39	67%	33%
40–44	66%	34%
45–49	64%	36%
50–54	64%	36%
55–59	63%	37%
60–64	63%	37%
65–69	63%	37%
70–74	63%	37%
75–79	65%	35%
80–84	68%	32%
85–89	72%	28%

### Disability-adjusted life year estimation

The DALY is the metric used to quantify the burden of a disease. DALYs are a combination of YLD and years of life lost (YLL) due to premature death [7]. In burden of disease studies, LBP is assumed not to carry any mortality, hence estimates of YLD are equivalent to DALY [13].

Considering the different severity proportions of LBP (with and without leg pain) the cases in each sub-group were estimated. The corresponding YLD for each sub-group and severity were then calculated by multiplying the number of prevalent cases with the respective disability weights. The total YLD was calculated as the sum of the health state specific YLD. The same principle was applied to calculate the YLD for each age-sex category. We used 100,000 Monte Carlo simulations to propagate the uncertainty in the prevalence estimates and disability weights. Resulting probability distributions were summarized by their mean and a 95% uncertainty interval (UI) defined as the distribution’s 2.5th and 97.5th percentile. The uncertainty in the age and sex specific prevalence estimates was characterized as a *Beta*(*x*, *n* − *x*) distribution, with *x* corresponding to the number of individuals with LBP in the age-sex group, and *n* to the corresponding total number of individuals. The uncertainty in the disability weights was characterized by fitting a Beta distribution to the disability weights’ mean and 95% confidence interval, using the beta Expert function in the R prevalence package [14].

Comparisons of point estimates were made to the LBP DALYs reported by the GBD 2017 study for Malta for the year 2015, and the number of prevalent cases were assessed against the 95% UIs [4]. All estimates are presented by five-year age-group and sex.

## Results

The prevalence of LBP with daily activity limitations was 6.4% (95% UI 5.7–7.2) (5.6% [95% UI 4.6–6.6] for males; 7.3% [95% UI 6.2–8.4] for females), corresponding to a total of 23,649 Maltese (95% UI 20,974–26,463) suffering from LBP (10,202 [95% UI 8475–10,089]) for males; 13,447 [95% UI 11,440–15,576]) for females). The burden of LBP among the Maltese population was 716 (95% UI 558–896) DALYs per 100,000 in a year, which corresponds to a total of 2633 (95% UI 2053–3298) healthy life years lost in a year.

**Fig. 1:**
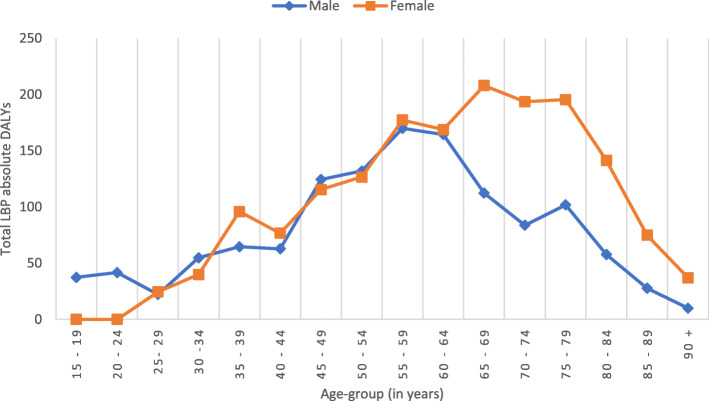
Distribution of low back pain disability adjusted life years by sex and age-groups

There were clear differences in how LBP affected males and females. It was observed that females experienced a higher LBP burden (739 [95% UI 575–927] DALYs per 100,000) than males (693 [95% UI 541–867] DALYs per 100,000). When stratified by age, DALYs increased with increasing age-groups (Fig. 1). Females exhibited the highest burden between 65 to 69 years while the males’ highest burden was between 60 to 64 years.

**Fig. 2:**
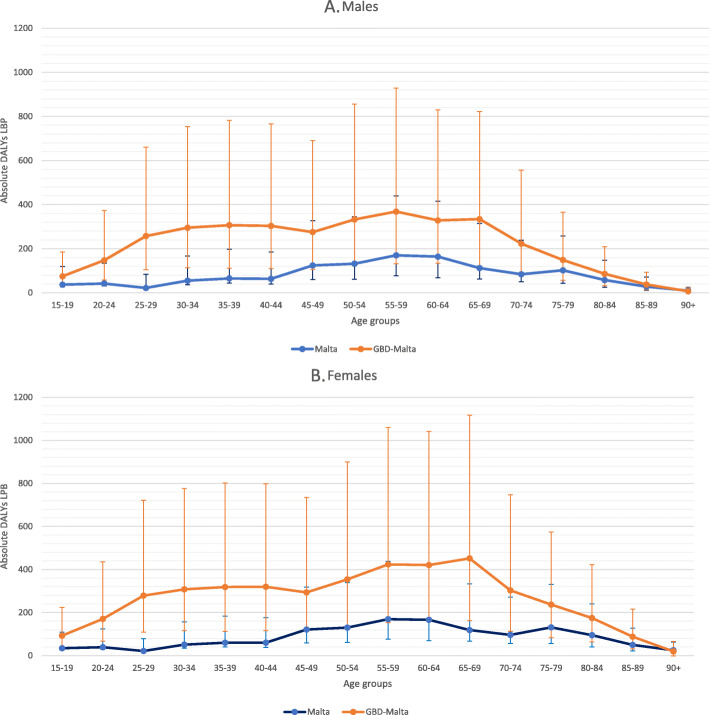
Comparisons of low back pain disability adjusted life years with uncertainty of the intervals between the current Malta study to the GBD 2017 study by age-group for (**a**) males; and (**b**) females

**Fig. 3:**
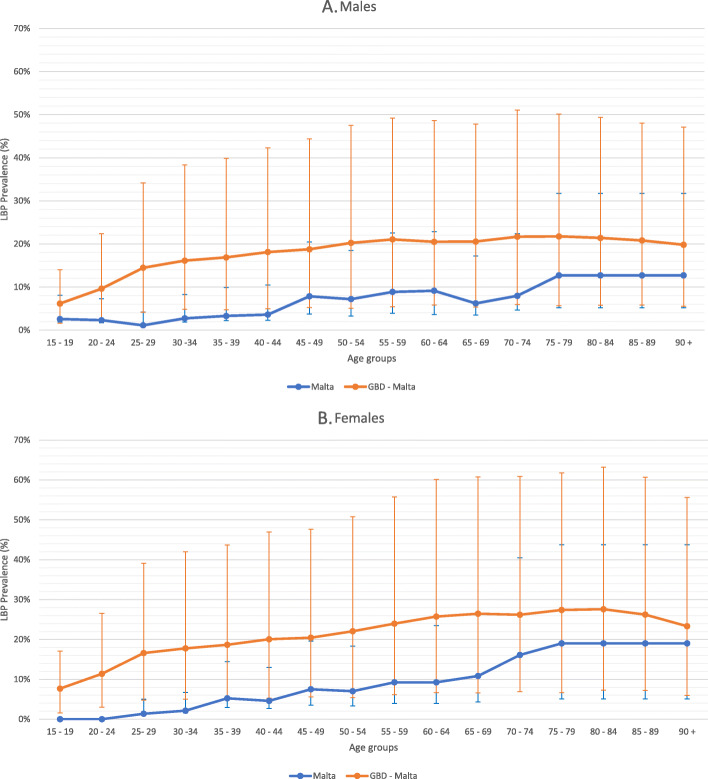
Comparisons of low back pain prevalence between the current Malta study to the GBD 2017 study by age-groupage-group for (**a**) males; and (**b**) females

Our LBP DALY estimates (716 [95% UI 558–896] DALYs per 100,000) were lower than those of the GBD 2017 study (1829 [95% UI 1300–2466] DALYs per 100,000) for Malta for the reference year 2015. The GBD 2017 estimates were found to be higher than our national estimation on age and sex stratification (Fig. 2). Considering the uncertainty intervals of the disability weights, it was observed that the GBD 2017 had much wider uncertainty intervals than the current study. Overlap of the uncertainty intervals for both studies was evident especially for the male population (Fig. 2). Our LBP prevalence estimates were much lower than those for the GBD 2017 study on age- and sex stratification (Fig. 3) and were found outside of the uncertainty intervals (UIs). The male 15 to 19 age-group had the lowest LBP prevalence relative difference with the GBD estimate 2.4 times greater than our estimates. On the other hand, a larger difference was observed in the 65 to 69 age-group for the males, where the GBD estimates were 3.3 times greater. A similar picture could be observed for the female population, with the 90 age-group having the lowest relative difference, whereby the GBD estimates were 1.2 times greater than our estimate. While the largest difference was in the 60 to 64 age-group, with the GBD estimates were 2.8 times greater. Our YLD per case estimate (0.11) was also found to be lower than that of the GDB 2017 study (0.14 YLD/case).

## Discussion

LBP is a public health concern which is affecting one in six Maltese. In fact, GBD reports that since 2007 LBP has been ranked the highest cause of disability as reflected by YLD in Malta [15]. Primary prevention strategies targeting LBP along with other chronic disease are priorities. These should follow a multisectoral approach, involving policymakers, public health officials, social services, health-care and management committees of workplaces [16].

Our study found that females experienced a higher LBP burden than males, similar to other European studies [17, 18]. When assessing sex differences across age-groups, Maltese females exhibited the highest burden discrepancy from males after the age of 60. Possible explanations for this finding could be that LBP is associated with the onset of menopause and to the reported fact that female obesity prevalence is higher than their male counterparts in this age-group [19]. There were vast differences in estimates of DALYs for Malta when our study’s DALYs were compared to the GBD 2017 study estimates for the reference year 2015. Both estimates followed the same disease model, severity proportions and disability weights for the year 2015. The differences were the case definition of LBP, the population data source and the prevalence of LBP. The case definition used in this study was stricter compared to the one used in the GBD 2017 with the inclusion of GALI, whereas in GBD 2017, LBP was defined as any type of LBP for at least 1 day. The GBD 2017 LBP data source originated from an older Malta-specific EHIS than the current study. Hence, the difference in case definition and data source could have led to the discrepancies in the prevalence rate. Using a more specific case definition and up-to-date survey data should enable a more accurate indication of the burden of LBP in Malta. On the other hand, a potential reason to the differences in the YLD per case is due to the variations in the proportion with leg pain vary by age. Of note, an overlap in DALY UIs was observed between this study and the GBD.

### Study limitations

The study population data source was obtained from a health interview survey. This consisted of self-reported weighed data that was representative of the whole Maltese population. Such data are subject to self-reporting bias and may have had an effect on the LBP prevalence. Since the definition used in this study included LBP that caused a limitation in the past 12 months, an element of underestimation might have been present. Individuals might not recall one episode that occurred a long time ago and then subsided. On the other hand, the prevalence of LBP beyond 75 years was assumed to be the same for all the following age-groups up till the age of 90 years. This may have led to an overestimation of the LBP in each of these age-groups since according to the GBD 2017 study, the LBP prevalence was on the decline as age progressed beyond 75 years. In order to try to correct for this overestimation, the cases of LBP were defined in accordance to the presence of disability limitations. However, of note, the LBP prevalence estimated within this study was found to be lower than the LBP prevalence reported by GBD 2017 study for Malta. Furthermore, the GBD study corrects for any overlap between LBP and injuries which was not feasible in our study since data was obtained from a survey. Consequently, a higher degree of overestimation of LBP prevalence could be present in our study. The duration of LBP was not considered in the definition nor in the analysis, but this is consistent with the model used in GBD 2017. It was assumed that the severity proportions of LBP and their corresponding disability weights, as reported by the GBD 2017 study, were the same in Malta. Differences in DALY estimates between the current study and the GBD 2017 study could be resulting from methodological differences such as mode of data collection, validation of instruments and representativeness of the samples [20].

### Implications for policy and practice

This study was based on nationally representative self-reported data, whilst it is not without limitation, but reflects the characteristics of the Maltese population. On the other hand, the GBD study based its estimates on different sources, not necessarily on local data or else based on old local data, which might be the reason for the observed discrepancies. It is important for policy makers to be able to relate findings from national surveys and other sources of estimates with those of the GBD study. If national population health surveys are going to be utilized it is essential to consider the elderly population since this population exhibits the highest disease burden. If the GBD study estimates are not consistent with national knowledge, there is real risk that policy makers will be challenged over which set of results they should relate to. It is therefore important that examples, such as those found in our study, are highlighted and worked through so that we can improve both national and GBD studies. If they are not, there is a major issue that burden of disease estimates could end up guiding incorrect decisions on policy direction or resource allocation. Hence, GBD should continue to integrate locally sourced data into their models in order to improve the DALYs estimates of each country. However, it is essential that the sourced data is up to date and there is the incorporation of country-specific severity distributions in the models. This can be achieved by local collaborations with clinicians and public health experts in order to generate proxy definitions for health states [21]. Local researchers should be continuously encouraged to be enrolled as GBD study collaborators, critically review the results of the country and ensure that up to date data sources are used for the national burden of disease estimates.

## Conclusions

LBP imposes a substantial burden on the Maltese population. The aetiology of LBP is multifactorial and hence it is suggested that a multisectoral preventive approach should be considered. The discrepancies observed between the national burden of disease and the global burden of disease study put forward the recommendation of integration of updated locally sourced data into the GBD model and increase the collaborations with local researchers. This would improve the DALY estimations for each country.

## Data Availability

The datasets analysed during the current study are not publicly available due to ownership being the Directorate of Health Information and Research, Ministry of Health, Malta, but are available from the corresponding author on reasonable request.

## Abbreviations

DALYs: Disability adjusted life years
EHIS: European Health Interview Survey
GALI: Global activity limitation indicator
GBD: Global burden of disease
LBP: Low back pain
UI: Uncertainty interval
YLD: Years lived with disability
YLL: Years of life lost
